# In Vitro Screening of Molecularly Engineered Polyethylene Glycol Hydrogels for Cartilage Tissue Engineering using Periosteum-Derived and ATDC5 Cells

**DOI:** 10.3390/ijms19113341

**Published:** 2018-10-26

**Authors:** Abhijith K. Kudva, Frank P. Luyten, Jennifer Patterson

**Affiliations:** 1 Department of Materials Engineering, KU Leuven, Kasteelpark Arenberg 44, Box 2450, 3001 Leuven, Belgium; akudva19@utexas.edu; 2 Prometheus, Division of Skeletal Tissue Engineering, KU Leuven, O&N 1, Herestraat 49, Box 813, 3000 Leuven, Belgium; frank.luyten@uzleuven.be; 3 Skeletal Biology and Engineering Research Center, KU Leuven, Herestraat 49, Box 813, 3000 Leuven, Belgium; 4 Oral and Maxillo-facial Surgery—Imaging & Pathology (OMFS-IMPATH), KU Leuven, Kapucijnenvoer 7 block a, Box 7001, 3000 Leuven, Belgium

**Keywords:** polyethylene glycol, ATDC5, periosteum-derived mesenchymal stem cells, chondrogenesis, hydrogels, matrix metalloproteinase

## Abstract

The rapidly growing field of tissue engineering and regenerative medicine has brought about an increase in demand for biomaterials that mimic closely the form and function of biological tissues. Therefore, understanding the cellular response to the changes in material composition moves research one step closer to a successful tissue-engineered product. With this in mind, polyethylene glycol (PEG) hydrogels comprised of different concentrations of polymer (2.5%, 4%, 6.5%, or 8% (*w*/*v*)); different protease sensitive, peptide cross-linkers (VPMSMRGG or GPQGIWGQ); and the incorporation or lack of a peptide cell adhesion ligand (RGD) were screened for their ability to support in vitro chondrogenesis. Human periosteum-derived cells (hPDCs), a mesenchymal stem cell (MSC)-like primary cell source, and ATDC5 cells, a murine carcinoma-derived chondrogenic cell line, were encapsulated within the various hydrogels to assess the effects of the different formulations on cellular viability, proliferation, and chondrogenic differentiation while receiving exogenous growth factor stimulation via the medium. Through the results of this screening process, the 6.5% (*w*/*v*) PEG constructs, cross-linked with the GPQGIWGQ peptide and containing the RGD cell binding molecule, demonstrated an environment that consistently supported cellular viability and proliferation as well as chondrogenic differentiation.

## 1. Introduction

The classical combination of cells, biomaterial scaffolds, and biomolecules is a main premise of tissue engineering strategies being explored to regenerate, repair, or replace compromised tissues and organs resulting from trauma, disease, or everyday wear and tear [1]. This approach first entails the isolation of tissue appropriate cells, followed by the expansion of the cells to the desired amount, their combination with biomaterial scaffolds and/or stimulatory factors, additional in vitro culture, and finally the implantation of the tissue-engineered construct into the host’s body to help heal the damaged tissue. Biomaterials are of particular importance in this process as they provide biochemical or mechanical cues to the cells in the tissue-engineered construct. These characteristics can also facilitate the reduction of the host response and improve tissue regeneration. An ideal biomaterial based scaffold should be temporary in nature and should support the initial stages of de novo tissue formation. One approach in scaffold design is to imitate aspects of the biological tissue to be regenerated, for example by trying to replicate the local extracellular matrix (ECM) [2]. The ECM is a sophisticated network of proteins and polysaccharides in the interstitial space that provides structure to the tissue; supports cell signaling; and influences the transport, sequestration, and presentation of biochemical cues such as growth factors. The composition of the ECM is highly tissue-specific, meaning that each type of tissue has its own ECM comprised of different proteins, polysaccharides, and signaling molecules that are vital for that particular tissue’s morphogenesis.

In that regard, several decades of research have been conducted emphasizing the use of biomaterials, more specifically hydrogels, for cartilage tissue engineering and regeneration [3]. Hydrogels are 3D hydrophilic networks that can imbibe large amounts of water, which is a major component of the native cartilage ECM [4], and can further be tailored to reproduce other chemical and mechanical properties of cartilage tissue. In addition, they can permit suitable nutrient and waste transportation, which is crucial for cell growth and survival. Hydrogels for tissue engineering applications can be designed from materials that are natural, synthetic, or a combination of both [5,6]. For the research reported herein, polyethylene glycol (PEG), a synthetic polymer, is used as the base material for the hydrogels. Although PEG is relatively inert biologically, it can be combined with bioactive components such as matrix metalloproteinase (MMP)-sensitive peptide linkages or cell adhesion ligands, and these systems have demonstrated the engineered biological activity and proved advantageous for tissue engineering applications [7]. PEG hydrogels fabricated using different chemistries have been extensively studied, and the influence of composition on the controlled variation of properties such as degradation rate, stiffness, and bioactivity have previously been demonstrated. The cross-linking density and mechanical properties of PEG hydrogels can be easily manipulated not only via the amount of cross-linker used but also by altering the concentration or structure of the PEG macromer [8,9]. For example, for cartilage tissue engineering, degradable PEG di-methacrylate (PEG-DM) hydrogels combined with a degradable poly(lactic-acid)-PEG-poly(lactic-acid) copolymer led to increased collagen type II synthesis and a more homogenous distribution of glycosaminoglycans (GAGs) produced by encapsulated bovine chondrocytes [10]. Further, PEG hydrogels with lower cross-linking densities have given rise to enhanced ECM production by both chondrocytes [10,11] and mesenchymal stem cells (MSCs) [12].

For the scope of this work, we aimed to test PEG hydrogels formed via the step growth mechanism of the Michael-Type addition reaction. The Michael-Type addition reaction involves nucleophilic addition to unsaturated electrophiles. To form the PEG hydrogel network, multi-arm, end-functionalized PEG macromers, such as vinyl sulfone end-functionalized PEG (PEG-VS), are cross-linked with peptide protease substrates that contain cysteine residues with reactive thiol groups (thiolates) at either end [13]. This approach is attractive as the reaction takes place at near physiological pH and temperatures and has been demonstrated to be selective for the thiol groups in the cross-linker molecules compared to biological amines [14]. Further, peptides containing greater than two reactive groups can be incorporated to form elastically-active network chains as part of a cross-linked 3D structure [13]. The 3D network that is formed can be varied based on several factors. The size of the PEG macromer (*M*_w_), the precursor concentration, and the number of end-functionalized branched arms present on the PEG play an important role in network formation and the properties of the resulting hydrogels. The mass swelling ratio is one property of hydrogels that provides information on the structure of the cross-linked network that is formed, including mesh size [15]. For these Michael-Type addition hydrogels, it has been demonstrated that, by increasing the *M*_w_ of the PEG, the equilibrium mass swelling ratios of the cross-linked networks increased irrespective of the dithiol cross-linker used [7,16]. Furthermore, as the concentration of the precursor solution increased prior to cross-linking, the resulting hydrogels’ equilibrium mass swelling ratio decreased, which was concluded to be a result of the intramolecular reactions being favored at low precursor concentrations whereas intermolecular cross-links were favored at high concentrations [16]. At the same time, it was shown that the elastic moduli of the hydrogels increased with increasing PEG concentration, irrespective of the *M*_w_, although the hydrogels formed from macromers with higher *M*_w_ displayed a lower elastic modulus at the same precursor concentration [13]. Moreover, for degradable hydrogels, as the *M*_w_ of the PEG macromer increased, the initial swelling ratio increased along with the apparent rate of degradation due to the increase in water content [16]. Similar MMP sensitive PEG-VS (4-arm, 20 kDa) hydrogels to those used herein have been characterized in previous studies and have displayed an average mesh size of approximately 0.025 µm and a storage modulus of close to 300 Pa for the composition of 10% (*w*/*v*) macromer [17].

In addition to the PEG macromer playing a role in the hydrogel properties, the dithiol peptide cross-linker utilized also has an impact. For instance, by altering the local electrostatic environment of the thiol by increasing its proximity to an additional charged amino acid and decreasing its dissociation constant (*pKa*), the gelation rate increased [13]. Moreover, the sequence of the particular amino acids present in the degradable peptide cross-linker had an influence on the overall degradation rate of the hydrogel [7,18]. The kinetic parameters (*K*_m_ and *k*_cat_) determined via Michaelis–Menten analysis of the degradation of individual peptide cross-linkers by specific MMPs correlated well with the degradation rate of hydrogels formed with these protease substrates by the same enzymes [18,19]. To build upon past research, herein we have specifically varied the composition of the PEG hydrogels through the use of different amounts of PEG (2.5%, 4%, 6.5%, and 8% (*w*/*v*)); different peptide sensitive cross-linkers (GPQGIWGQ vs. VPMSMRGG), varying in their degradation rates [19]; and lastly, different amounts of the cell binding motif, RGD, covalently bound in the PEG hydrogels. These various hydrogels were screened by encapsulating either human periosteum-derived cells (hPDCs), which are primary MSC-like cells, or ATDC5 cells, a cell line derived from mouse carcinoma.

The cells in the periosteum are a source for the osteoblasts that enable appositional bone growth during bone homeostasis and, in combination with osteoclasts, help the remodeling of cortical bone [20]. The inner cambium of the periosteum also serves as a main source of progenitor cells during fracture healing [20], and the PDCs harvested and expanded from this tissue have demonstrated high proliferative capacity and multilineage potential [21,22,23]. PDCs have also displayed osteochondrogenic potential in vivo, when seeded on 3D calcium phosphate scaffolds [24]. Additionally, hPDCs have shown chondrogenic potential when cultured in vitro in micromass conditions [25] as well as when encapsulated within 3D hydrogels [26], thus making them an attractive cell source for skeletal tissue engineering. On the other hand, as with other adult stem cells, harvesting of PDCs also has shortcomings. Although harvesting tissue from this region could alter tissue homeostasis, PDCs possess a high proliferative capacity and hence a relatively small biopsy would be required. Furthermore, the harvest of small tibial periosteal flaps has been FDA approved for autologous chondrocyte implantation (ACI) [27].

While MSCs and PDCs would seem ideal for use in tissue engineering strategies, some of the drawbacks associated with the use of primary cells, such as donor availability and/or donor variability, have prompted in vitro investigation with more reproducible cell lines. Immortalized cell lines demonstrate advantages such as infinite, homogenous proliferation as well as consistent differentiation as they are composed of a pure population of cells [28]. Furthermore, cell lines are easy to use and cost effective, and they avoid the ethical concerns that accompany the use of primary animal or human cells. These characteristics make cells lines a very attractive source when evaluating biomaterials as they help to make in vitro testing facile and reproducible.

The cell line used in this study, ATDC5, was derived from AT805 mouse teratocarcinoma fibroblastic cells and established by Atsumi et al. as a promising in vitro model to study chondrogenesis [29]. In 2D culture, these cells exhibited a fibroblastic cellular phenotype during the growth phase, and the subsequent introduction of insulin caused the cells to continue to grow past confluence and form aggregates resembling cartilaginous nodules with chondrogenic specific proteoglycan production [29]. Since then, additional in vitro studies have highlighted the value of ATDC5 cells for studying chondrogenesis. For instance, Shukunami et al. have shown that ATDC5 cells undergo a chondrogenic process in a sequential manner similar to chondrocyte differentiation [30,31]. During this chondrogenic process, ATDC5 cells have also displayed the Indian hedgehog (Ihh) [32] and parathyroid hormone (PTH)/parathyroid hormone receptor protein (PTHrP) [31] signaling pathways, which are key signaling pathways that determine the fate of chondrocytes. ATDC5 cells in micromass, pellet, or aggregate cultures have been used to investigate the influence of various factors in cartilage tissue engineering strategies, for example, the effects of hypoxic conditions on chondrogenesis [33,34]. Furthermore, ATDC5 aggregates that were chondrogenically stimulated in vitro and implanted in vivo using a 3D scaffold showed signs of ectopic bone formation [35]. ATDC5 cells have also been cultured on synthetic polymer hydrogels with various charge densities [36]. By seeding ATDC5 cells on either a neutral poly(dimethylacrylamide) (PDMAAm) hydrogel or a negatively charged poly(2-acrylamido-2-methyl-1-propanesulfonic acid) (PAMPS) hydrogel, chondrogenic differentiation was induced in insulin-supplemented differentiation medium and in insulin-free maintenance medium, respectively [36]. Another study displayed high ATDC5 metabolic viability and proliferation when they were encapsulated within injectable, hyaluronic acid/PEG hydrogels [37].

Hence, in this study, we aim to harness the potential of hPDCs along with the well characterized ATDC5 chondrogenic cell line to screen the different PEG hydrogel formulations described above for their ability to support progenitor cell proliferation as well as chondrogenesis. For proliferation, the change in DNA content within the cell-laden constructs was measured, whereas to assess chondrogenic differentiation, the cellular glycosaminoglycan (GAG) production was analyzed, along with qualitative histological staining for GAG and collagen. Although we have previously demonstrated the ability of the 6.5% (*w*/*v*) PEG hydrogels functionalized with GPQGIWGQ and RGD peptides to support proliferation and chondrogenesis of hPDCs [26] as well as redifferentiation of 2D expanded human articular chondrocytes [38], the novelty of the present study is the more extensive variation of the PEG hydrogel compositions to further explore the influence of material properties on cell behavior as well as the evaluation of the behavior of ATDC5 cells in this hydrogel system.

## 2. Results and Discussion

### 2.1. Screening Using hPDCs Encapsulated in PEG Hydrogels

#### 2.1.1. Hydrogel Composition Affects the Encapsulated hPDCs’ Viability and Proliferation when Cultured in Growth Medium

**Figure 1:**
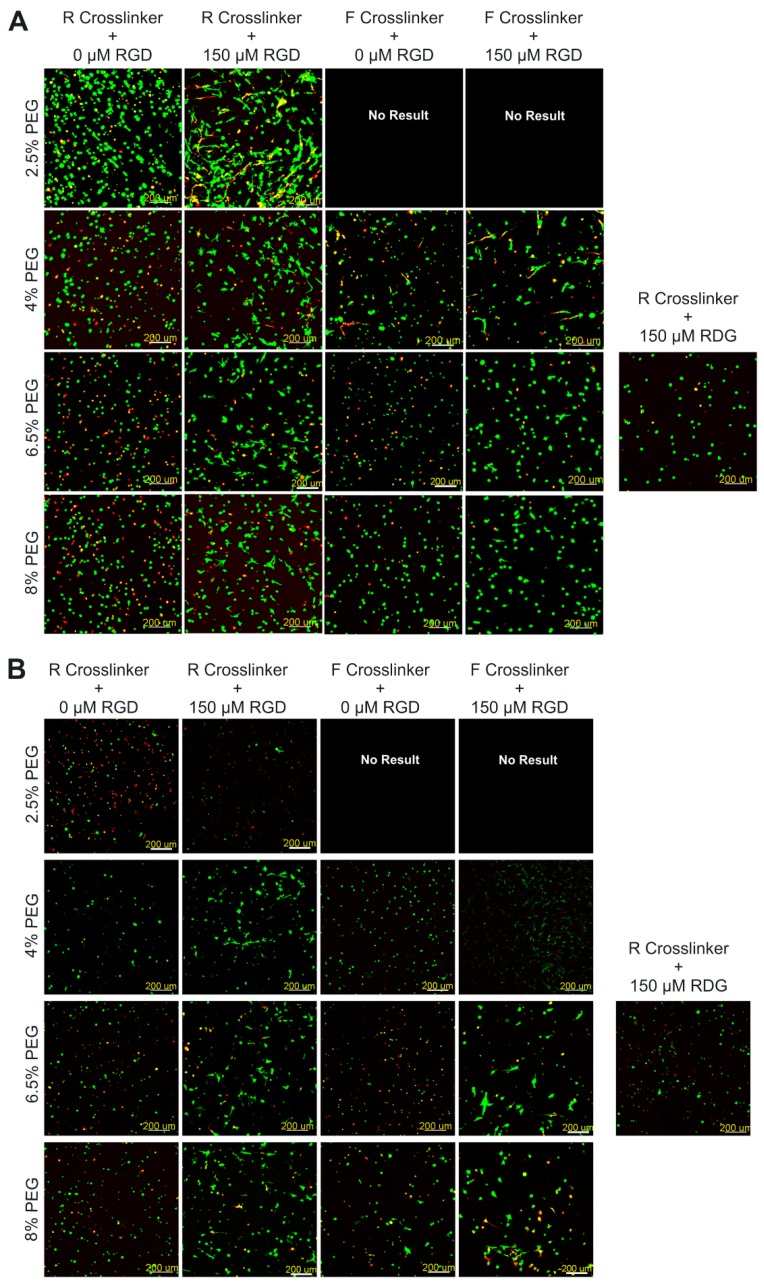

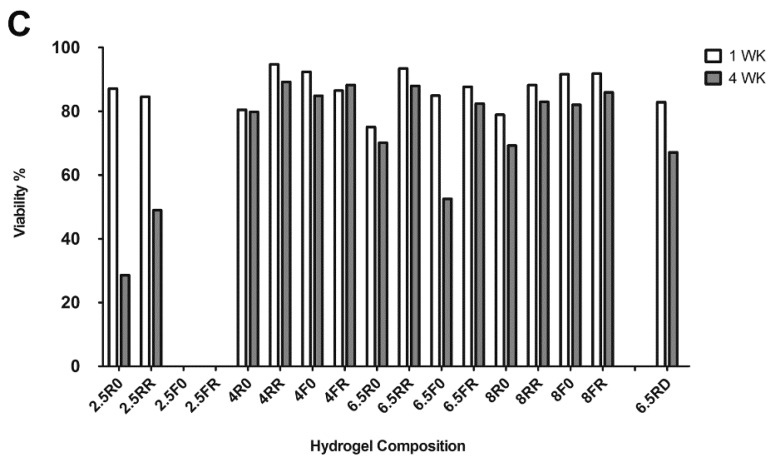
Human periosteum-derived cell (hPDC) viability within PEG-VS hydrogels made with different concentrations of macromer, different protease sensitive cross-linkers (R or F), and with or without the cell binding motif RGD or scrambled RDG after 1 week (**A**) and 4 weeks (**B**) of in vitro culture in GM. Representative images of Live/Dead staining with calcein AM (green, live cells) and ethidium homodimer-1 (red, dead cells). Confocal images are depicted as the maximum intensity of a 500 μm z-stack that was acquired using a 10× objective (scale bars = 200 μm). (**C**) IMARIS software based quantification of viability percentages of cells encapsulated in polyethylene glycol (PEG) hydrogels (*n* = 1 independent sample; values depicted are the averages of two different measured areas within one sample).

Molecularly engineered hydrogels (see Table 1 for a summary of the compositions as well as nomenclature used) were made with the concentration of PEG ranging from 2.5% to 8% (*w*/*v*), variation in the type of peptide cross-linker incorporated, and the addition or lack of the cell binding ligand, RGD. In particular, the protease-sensitive peptide cross-linker varied in sequence between a substrate derived from type I collagen (GPQGIWGQ, referred to as the regular degrading cross-linker or R throughout) and a faster degrading cross-linker (VPMSMRGG, referred to as F throughout) [18,19]. In previous studies, the R cross-linker had measured *k*_cat_ values of 0.65 ± 0.13 s^−1^ and 2.17 ± 0.16 s^−1^ with respect to MMP-1 and MMP-2 [19]. Additionally, it was shown that PEG hydrogels composed of the R cross-linker continued to persist longer than 10 days when incubated with MMP-1 but degraded after 4 days when incubated with MMP-2 [19]. Furthermore, the F cross-linker had significantly higher *k*_cat_ values (5.25 ± 0.95 s^−1^ for MMP-1 and 4.82 ± 0.79 s^−1^ for MMP-2), when compared to the R cross-linker. The *k*_cat_ value of the F cross-linker was also 3.79 ± 0.62 times higher than that of the R cross-linker for the enzyme plasmin [18]. Moreover, the hydrogels containing the F cross-linker also degraded significantly faster in the presence of MMP-1 and MMP-2 (~1.5 and 2 days, respectively), when compared to the hydrogels comprised of the R cross-linker treated with these enzymes [19]. After 1 week of in vitro culture in growth medium (GM), the hPDCs encapsulated within the various hydrogel compositions displayed a good level of viability (Figure 1A), with no composition displaying a viability percentage of less than 75% (Figure 1C). Already at this time point, the hydrogels with the lowest concentration and the faster degrading cross-linker (2.5F0 and 2.5FR) were too degraded to be further handled, and thus these formulations were not able to be evaluated throughout the rest of the study. When observing the viability of the hPDCs in the PEG hydrogels after four weeks of in vitro culture in GM (Figure 1B), the cells in the hydrogels without any RGD demonstrated a drop in their viability, sometimes displaying a cellular viability percentage as low as approximately 50% (Figure 1C). These results were as expected since hPDCs are adherent cells. The binding of integrins on the surface of adherent cells to RGD leads to a cascade of signaling pathways that are important for their survival [39].

Further, although the calcein stain in the Live/Dead assay is not specific for the cytoskeleton, its distribution in the cytoplasm displays the cellular morphology. From these images (Figure 1), it is also clearly visible that the components of the hydrogels affected the shape of the hPDCs. At both time points, the cells in the hydrogels containing 150 µM RGD (2.5RR, 4RR, 4FR, 6.5RR, 6.5FR, 8RR, and 8FR) displayed a more spread morphology than the cells in the hydrogel formulations without RGD (2.5R0, 4R0, 4F0, 6.5R0, 6.5F0, 8R0, and 8F0). Moreover, the incorporation of a peptide with the scrambled sequence, RDG, did not lead to a spread morphology of the hPDCs, which further confirms the specificity of the peptide functionalization with RGD. Additionally, after only one week in culture, hPDCs in the hydrogels with the lowest concentration of PEG (2.5RR) already seemed to have a much more elongated shape than hPDCs in the 4RR, 6.5RR, and 8RR hydrogels. One reason could be because the lower percentage of PEG used leads to lower hydrogel stiffness and the hPDCs sense a different mechanical rigidity, which in turn affects their shape. It has been well demonstrated that the amount of macromer used and the modulus of the construct are inversely related [11] and hence can affect the cellular response. Previous work conducted with similar PEG-VS hydrogels comprised of an adhesion ligand and cross-linked with MMP sensitive peptides or nondegradable dithiol cross-linkers have demonstrated a storage modulus (G’) ranging from 0.3 to 2 kPa [17,40,41,42]. More specifically, 4-arm 20 kDa PEG-VS hydrogels containing RGD and the same peptide sensitive cross-linkers as used herein showed an increase in G’ from approximately 0.5 to 1.5 kPa as the macromer concentration increased from 3.5% to 5% (*w*/*v*), respectively [42]. Changing the peptide sequence in the cross-linker from GPQGIWGQ (R cross-linker) to VPMSMRGG (F cross-linker) did not change G’ [42]. These values corresponded well with the storage modulus of 1.2 ± 0.13 kPa that our group had measured for 6.5% (*w*/*v*) PEG-VS hydrogels that were cross-linked with dithiothreitol (DTT) [43]. Furthermore, it has also been shown that naïve stem cells such as MSCs, due to their plasticity, respond differently in lineage specification and their morphology dependent on the microenvironment of the matrix presented to them [44].

**Figure 2:**
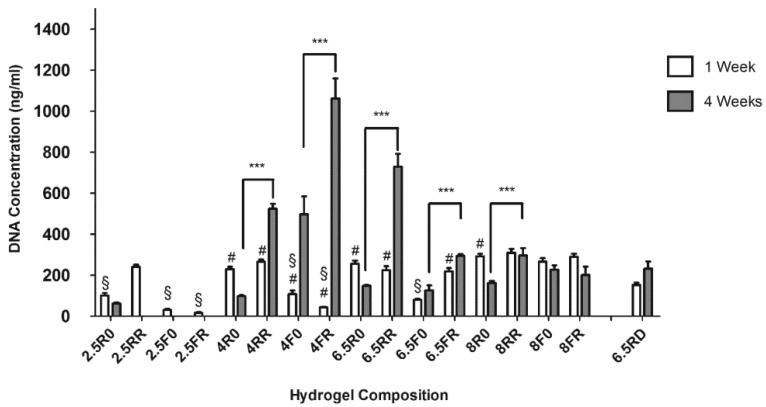
DNA content of cell-laden PEG hydrogels cultured in GM in vitro over time varying in the percentage of macromer, the cross-linker type, and the incorporation or lack of the cell binding motif, RGD, or scrambled peptide, RDG. Results are presented as mean ± SD (*n* = 3; # *p* < 0.001 when comparing the hydrogel composition at 1 week to its 4 week counterpart; § *p* < 0.01 compared to 1 week DNA content of unmarked hydrogels; *** *p* <0.001 when comparing otherwise similar hydrogels with and without RGD at 4 weeks).

Next, the DNA content of the various cell-laden hydrogels was analyzed after one week and four weeks of in vitro culture in GM (Figure 2). At one week, many of the hydrogels displayed a similar DNA content (insignificant *p*-values), with the exception of some compositions, namely 2.5R0, 2.5F0, 2.5FR, 4F0, 4FR, 6.5F0, and 6.5FR, showing a significantly reduced DNA content (*p* < 0.01). These compositions tended to be the ones with the faster degrading cross-linker and/or the lower concentrations of PEG. Nonetheless, after four weeks of culture, the DNA content in the RGD containing PEG hydrogels, specifically in the 4% and 6.5% (*w*/*v*) constructs, was significantly higher (*p* < 0.001) than not only the DNA content in their one week counterparts but also the DNA amounts in their counterparts without RGD at week 4 (*p* < 0.001). This indicates that time and hydrogel composition, especially the cell binding motif, play a crucial role in cell proliferation for hPDCs encapsulated within these PEG constructs. After one week, the 2.5% (*w*/*v*) PEG hydrogels, as mentioned before, were already swollen and weak, and hence their data should be interpreted with caution. On the other hand, although the 8% (*w*/*v*) PEG hydrogels demonstrated the capability to support hPDC viability over time, these hydrogels did not display any significant difference in their DNA content over time, except in the 8R0 hydrogels, where there was a significant drop in DNA content after four weeks (*p* < 0.001). This lack of cell proliferation in the 8% (*w*/*v*) PEG hydrogels could be due to the higher percentage of PEG leading to an increase in cross-linking density and stiffness and a decrease in mesh size. Previous work with PEG hydrogels has shown that doubling the concentration of PEG macromer led to an almost two-fold decrease in mesh size [9]. In addition, it has been previously demonstrated, with chondrocytes [45] as well as fibroblasts [46], that increasing mesh size supported cellular proliferation. Interestingly, many of the constructs without RGD displayed a significant drop in DNA content after four weeks (*p* < 0.001). The lack of RGD, combined with the slower degrading cross-linker, seemed to have a negative impact on hPDC proliferation, as the 4R0, 6.5R0, and the 8R0 groups all demonstrated lower DNA content at four weeks compared to the DNA content of these groups at one week. The drop in DNA content of the hPDCs in the R0 hydrogels over time appears to be reproducible, as we have previously reported [26].

#### 2.1.2. GAG Production of hPDCs Encapsulated in PEG-VS Hydrogels Increases over Time when Cultured in Chondrogenic Differentiation Medium

**Figure 3:**
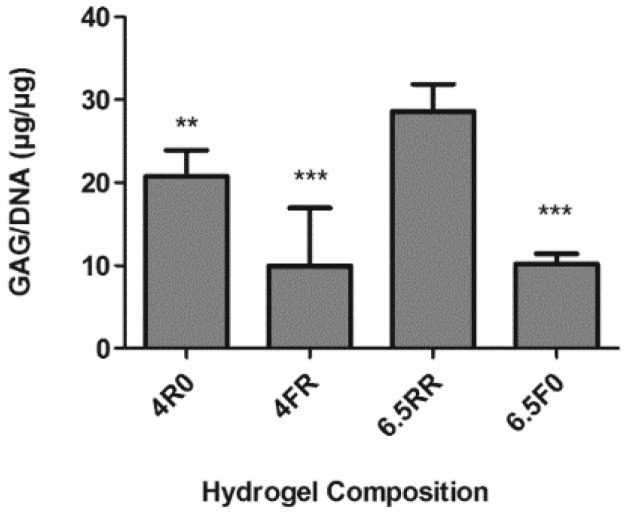
GAG/DNA content of selected PEG hydrogels after four weeks of in vitro chondrogenic stimulation in 4C chondrogenic medium. Hydrogel formulations were chosen based on previous proliferation data and design of experiments (DoE)-based software. Results depicted as mean ± SD (*n* = 3; Student’s *t*-Test ** *p* < 0.01, *** *p* < 0.001 when compared to 6.5RR composition).

In screening experiments such as this, it can quickly become infeasible to test all of the possible combinations of variables. To address this limitation, the design of experiments (DoE) approach is a powerful tool that allows the simultaneous evaluation of multiple variable/parameters in an efficient manner [47]. The proliferation data reported in the previous section were used with JMP software to create a fractional factorial design with three factors (PEG%, RGD concentration, and cross-linker type) and two levels. Because the 2.5% and 8% (*w*/*v*) PEG constructs did not perform well in the proliferation experiment, they were excluded from the DoE analysis. Based on the 3-factor, multilevel DoE results, the 4R0, 4FR, 6.5RR, and 6.5F0 hydrogel compositions were selected to conduct a preliminary study of chondrogenic differentiation of hPDCs within the PEG hydrogels when cultured in 4C chondrogenic medium, which was measured by determining the glycosaminoglycan (GAG) production by the cells. The GAG/DNA content (Figure 3) of these groups demonstrated that the 6.5RR hydrogel formulation had a significantly higher GAG/DNA content than the other hydrogels after four weeks of culture. Based on the previous proliferation results and this preliminary chondrogenic differentiation study, it was concluded that the 6.5% (*w*/*v*) PEG hydrogels not only provided an environment to sustain hPDC proliferation but they also had the potential to support GAG production of the hPDCs when cultured in chondrogenic medium.

**Figure 4:**
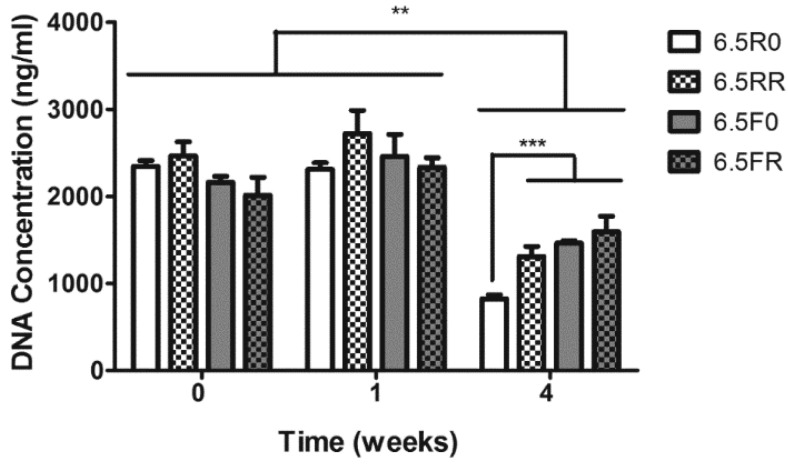
DNA quantification of encapsulated hPDCs within 6.5% (*w*/*v*) PEG hydrogels composed of two different types of cross-linkers (F or R) and functionalized with 150 µM or 0 µM RGD, when cultured in 4C chondrogenic medium. 6.5R0 = R cross-linker + 0 µM RGD; 6.5RR = R cross-linker + 150 µM RGD; 6.5F0 = F cross-linker + 0 µM RGD; 6.5FR = F cross-linker + 150 µM RGD. Results depicted as mean ± SD. (Two-way ANOVA; *n* = 3; *** *p* < 0.001; ** *p* < 0.01).

As the 6.5RR group was one of the best performing hydrogel compositions in both of the prior experiments, a further investigation of the chondrogenic differentiation of hPDCs when encapsulated in 6.5% (*w*/*v*) PEG hydrogels and cultured in chondrogenic 4C medium was performed. The hydrogels varied only in the protease sensitive cross-linker used and the presence or absence of RGD. Cell-laden constructs were cultured in GM for 24 h and then subsequently switched to chondrogenic 4C medium and cultured for 1 and 4 weeks. When looking at the DNA content (Figure 4) of these 6.5% (*w*/*v*) PEG hydrogels containing cells, at 0 weeks there was no difference in DNA content, implying that the hPDCs were initially encapsulated equally in the different hydrogels. At 1 week, there was no significant increase in the DNA content observed within these hydrogels that were cultured in 4C chondrogenic medium. However, at 4 weeks, the DNA content significantly dropped across all hydrogel formulations (*p* < 0.01). Moreover, in a similar trend as seen in the proliferation experiment (Figure 2), the 6.5R0 and 6.5F0 hydrogels displayed lower DNA content compared to their RGD containing counterparts, 6.5RR and 6.5FR, respectively. Additionally, the 6.5R0 construct displayed the lowest DNA content compared to the rest of the hydrogel formulations (*p* < 0.001). This drop in DNA content over the 4 weeks can possibly be attributed to the cell seeding density and/or the culture medium. The cells were encapsulated at a higher starting cell density than in the proliferation experiments reported in Section 2.1.1, and the cell-laden constructs were cultured in the 4C chondrogenic medium, which would favor differentiation over proliferation. Further, previous studies have reported that a higher cell density was not beneficial for proliferation since the cells tended to enter the quiescent phases of the cell cycle when cultured in conditions promoting differentiation [48].

**Figure 5:**
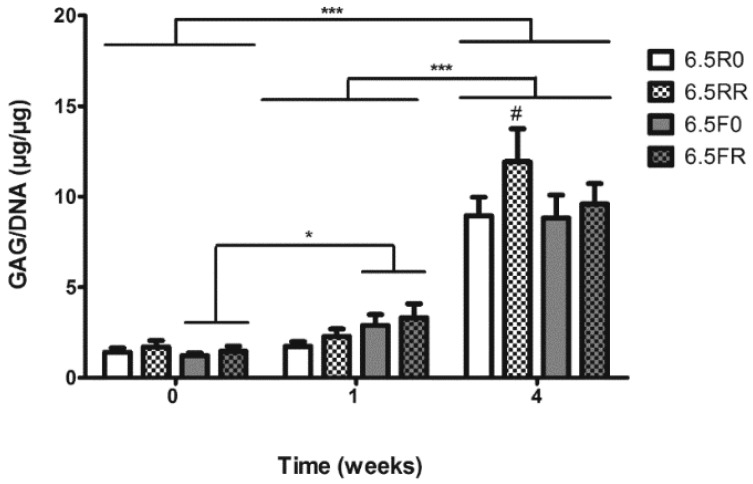
GAG/DNA content of encapsulated hPDCs within 6.5% (*w*/*v*) PEG hydrogels composed of two different types of cross-linkers (F or R) and functionalized with 150 or 0 µM RGD, when cultured in 4C chondrogenic medium. 6.5R0 = R cross-linker + 0 µM RGD; 6.5RR = R cross-linker + 150 µM RGD; 6.5F0 = F cross-linker + 0 µM RGD; 6.5FR = F cross-linker + 150 µM RGD. Results are depicted as mean ± SD. (Two-way ANOVA, *n* = 3; *** *p* < 0.001; * *p* < 0.05; # *p* < 0.05 when comparing to other hydrogel formulations at the particular time point).

The DMMB GAG assay showed very low amounts of GAG/DNA being produced at 0 weeks (Figure 5). Additionally, there was no significant difference observed among the hydrogel compositions at this time point. After 1 week of chondrogenic stimulation via the 4C medium, the hPDCs in the hydrogels with the F cross-linker displayed a significant increase in GAG/DNA content (*p* < 0.05), but the hPDCs in the hydrogels with the R cross-linker did not experience any significant change. After 4 weeks of in vitro culture, all of the hydrogel formulations saw a significant increase in GAG production by the hPDCs compared to the hPDCs in the hydrogels at 0 and 1 weeks (*p* < 0.001). At the 4 week time point, the GAG production of the encapsulated hPDCs in the hydrogels cross-linked with the R cross-linker and containing RGD (6.5RR) was significantly higher than in the other hydrogel formulations. This trend correlates well with the results obtained from the DoE experiment, where the hPDCs in the 6.5RR hydrogel demonstrated the highest GAG/DNA production after 4 weeks of culture, although the absolute amount was higher in the previous results (Figure 3). This variance could arise due to the utilization of different biological replicates as well as the use of primary cells. Additionally, when comparing the GAG/DNA in this study to another study conducted with hPDC micromasses incorporating growth factor releasing gelatin microspheres [49], the GAG accumulation per cell was higher with the PEG constructs. This could indicate that the GAG is being retained in the PEG matrix, which would be beneficial when forming a cartilaginous tissue-engineered construct.

Based on the results from both the proliferation and differentiation assays with hPDCs, it was concluded that the 6.5% (*w*/*v*) PEG hydrogels were well suited to carry out further experimental testing. Additionally, although the 2.5% (*w*/*v*) PEG hydrogels were deemed structurally unsound for the purpose of creating an in vitro cartilaginous construct, they could be an attractive choice as an injectable hydrogel for articular cartilage surface repair [50,51,52]. The low PEG macromer amount used could make it injectable via a syringe after hydrogel formation, as these PEG constructs with low macromer concentrations have been shown to possess lower moduli and lower stiffness [53] and thus could be deformed when passing through a syringe.

### 2.2. Screening Using ATDC5 Cells Encapsulated in PEG Hydrogels and Cultured in Chondrogenic Differentiation Medium

#### 2.2.1. Extent of ATDC5 Viability and Proliferation when Encapsulated within PEG Hydrogels and Cultured in Chondrogenic Differentiation Medium

**Figure 6:**
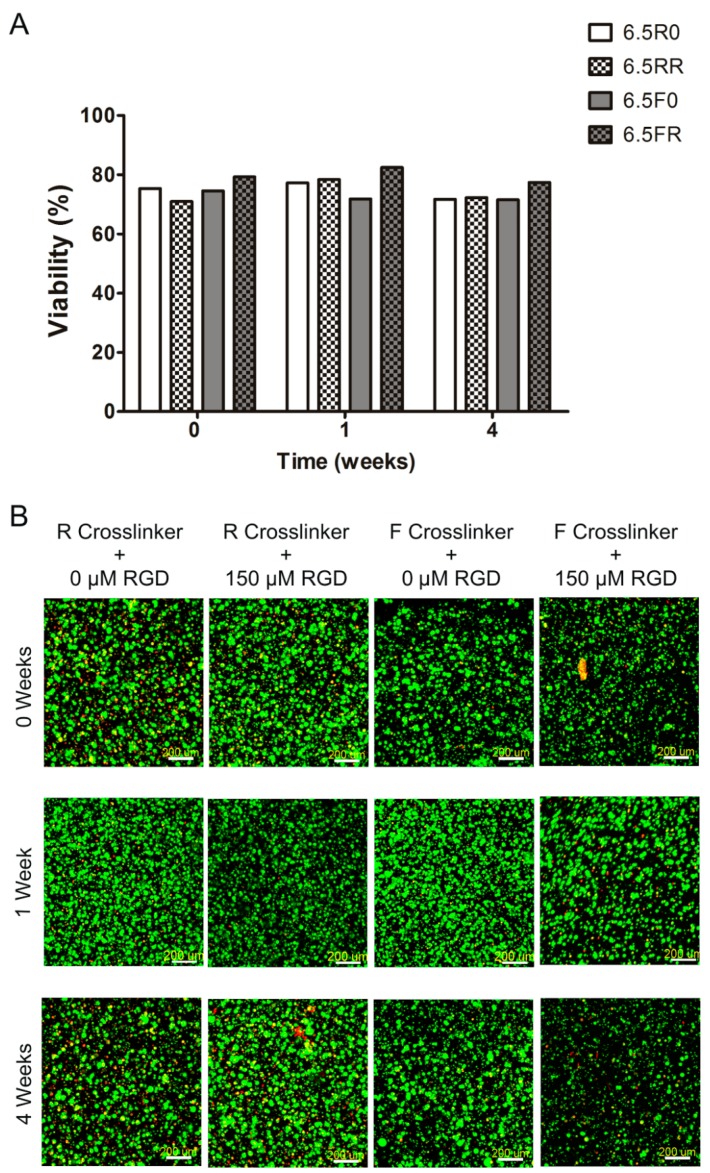
ATDC viability within protease sensitive 6.5% (*w*/*v*) PEG-VS hydrogels, with different cross-linkers (F or R) as well as with and without RGD, cultured over time. Constructs were examined at 0 weeks (24 h in MM) and 1 and 4 weeks in the 4C chondrogenic differentiation medium. 6.5R0 = R cross-linker + 0 µM RGD; 6.5RR = R cross-linker + 150 µM RGD; 6.5F0 = F cross-linker + 0 µM RGD; 6.5FR = F cross-linker + 150 µM RGD. (**A**) IMARIS software based quantification of viability percentages of ATDC5 cells encapsulated in PEG hydrogels (*n* = 1; values depicted are the averages of two different measured areas within one sample). (**B**) Representative confocal images of Live/Dead staining shown as the maximum intensity of a 500 µm z-stack where live cells are green and dead cells are red (scale bar = 200 µm).

As aforementioned, ATDC5 is a valuable cell line to test in vitro chondrogenesis. In this study, the use of a cell line instead of primary cells allowed us to perform additional assays, such as histology staining, to characterize the behavior of the cells within the protease sensitive PEG hydrogels when cultured in chondrogenic differentiation medium. Further, they allowed us to validate if the most promising hydrogel compositions identified in Section 2.1 show reproducible chondrogenesis with a different cell source. First, in the 6.5% (*w*/*v*) PEG hydrogels, the ATDC5 cells displayed a viability percentage of approximately 75% or higher at 0 weeks (Figure 6A). Moreover, as they continued to be cultured over 4 weeks in the 4C chondrogenic differentiation medium, they demonstrated no drop in viability for any of the different hydrogel constructs. This can be qualitatively observed in the images of the Live/Dead staining (Figure 6B), as all of the panels display a majority of green (live) cells as opposed to red (dead) cells. The viability results herein are similar to when ATDC5 cells were encapsulated within hyaluronic acid/PEG hydrogels formed via click chemistry, where the cells displayed a high viability after 7 days of culture in a maintenance medium composed of DMEM and F12 [37].

**Figure 7:**
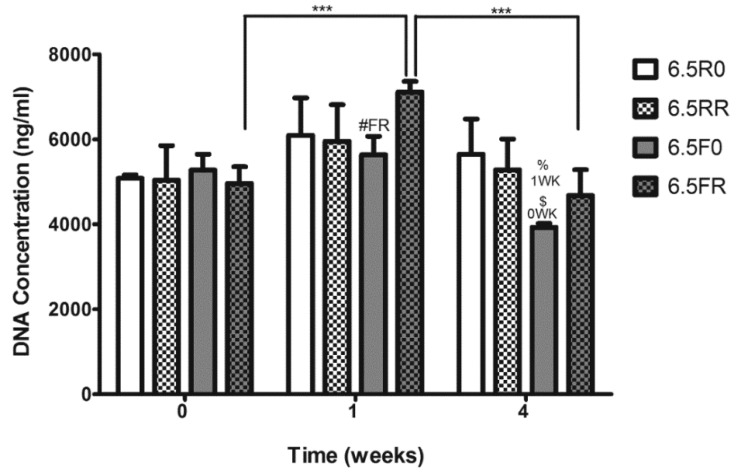
DNA quantification of ATDC5 cells encapsulated inside 6.5% (*w*/*v*) PEG hydrogels varying in the cross-linker type (F or R) and RGD content, and cultured over time in 4C chondrogenic medium. 6.5R0 = R cross-linker + 0 µM RGD; 6.5RR = R cross-linker + 150 µM RGD; 6.5F0 = F cross-linker + 0 µM RGD; 6.5FR = F cross-linker + 150 µM RGD. Results depicted as mean ± SD (Two-way ANOVA; *n* = 3; #FR *p* < 0.01 compared to FR formulation at 1 week; %1WK *p* < 0.001 compared to the hydrogel at 1 week with the same composition. $0WK *p* < 0.001 compared to the hydrogel at 0 week with the same composition; *** *p* < 0.001).

Following the evaluation of the viability of ATDC5 cells, the proliferation of these cells within these constructs, when cultured in 4C medium, was determined (Figure 7). At the 0 week time point, there was no significant difference in the DNA content of the different constructs. As with the results for hPDCs in Section 2.1.2, this lack in difference in DNA content demonstrates equal initial cell amounts inside the hydrogels. At 1 week, the hydrogels with the regular degrading cross-linker (6.5RR, 6.5R0) did not have a significant difference in DNA content compared to their respective counterparts at 0 weeks. However, the cells within the 6.5FR hydrogels displayed a significant increase in their DNA content (*p* < 0.001) when compared to cells in the same hydrogel formulation at 0 weeks, which was an indication that the cells were proliferating in this specific hydrogel composition. Furthermore, at this time point, the cells displayed significantly more DNA content in the 6.5FR hydrogels compared to the 6.5F0 construct (*p* < 0.01). Similar to 1 week, at 4 weeks the cells within both of the hydrogels with the R cross-linker did not demonstrate a change in DNA content, thus maintaining their cellular numbers over the entire culture time. After witnessing a significant increase in the DNA content of the 6.5FR group after 1 week of in vitro culture, the ATDC5 cells demonstrated a significant drop in DNA levels (*p* < 0.001). However, this drop was only down to the levels at 0 weeks, and no difference was observed between 0 and 4 weeks. Lastly, the 6.5F0 hydrogel displayed a significant drop (*p* < 0.001) in ATDC5 DNA content when compared to its cell-laden counterparts at 0 and 1 week.

#### 2.2.2. GAG and Collagen Production by ATDC5 Cells Encapsulated in Protease Degradable PEG-VS Hydrogels

**Figure 8:**
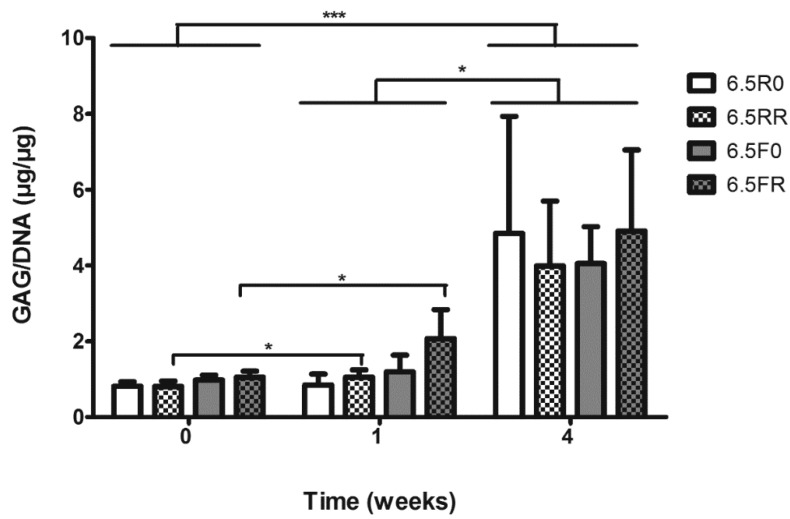
GAG/DNA content of ATDC5 cells encapsulated inside 6.5 (*w*/*v*) PEG hydrogels varying in the cross-linker type (F or R) and RGD content (0 or 150 µM), and cultured over time in 4C chondrogenic medium. 6.5R0 = R cross-linker + 0 µM RGD; 6.5RR = R cross-linker + 150 µM RGD; 6.5F0 = F cross-linker + 0 µM RGD; 6.5FR = F cross-linker + 150 µM RGD. Results depicted as mean ± SD (Two-way ANOVA *n* = 3; * *p* < 0.05; *** *p* < 0.001).

To examine the in vitro chondrogenic capability of ATDC5 cells in PEG hydrogels, a DMMB GAG assay and histology were used to quantitatively and qualitatively demonstrate the extent of chondrogenic differentiation taking place within the hydrogel constructs. The quantitative GAG assay displayed a significant increase in the production of GAG by ATDC5 cells within these hydrogels (Figure 8). At 0 weeks, before being switched to chondrogenic medium, the GAG production by the encapsulated cells was very low among all the hydrogel formulations. After one week of in vitro culture in the 4C medium, the RGD containing hydrogels, 6.5RR and 6.5FR, demonstrated a significant increase (*p* < 0.05) in GAG production by the encapsulated ATDC5 cells, compared to the cellular GAG production in their respective hydrogel formulations at 0 weeks. 6.5FR is the same hydrogel formulation that displayed a significant DNA increase after one week of culture. After four weeks of in vitro chondrogenic stimulation, all of the ATDC5 laden hydrogels displayed a significant increase in GAG/DNA content not only compared to one week (*p* < 0.05) but also compared to 0 weeks (*p* < 0.001). However, at 4 weeks, there was no difference among the various hydrogel formulations.

**Figure 9:**
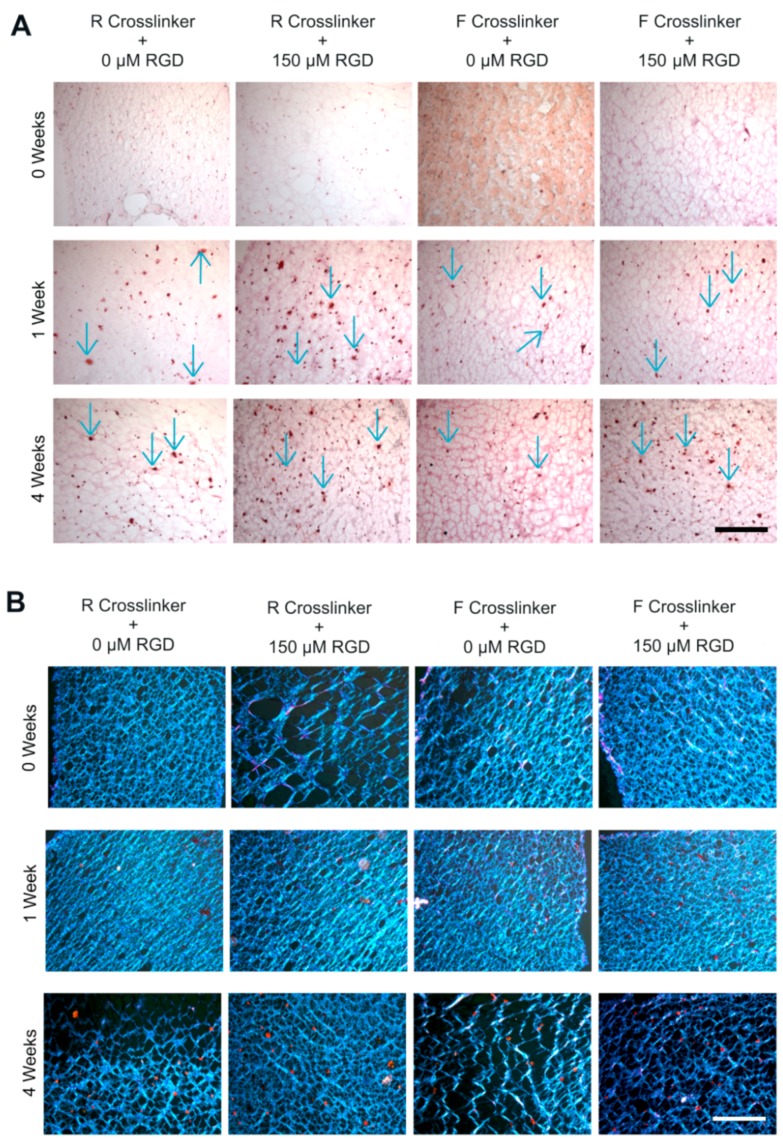
Representative images of histological staining of ATDC5 cells encapsulated in 6.5% (*w*/*v*) PEG hydrogels. (**A**) Brightfield images of safranin-O stained sections for visualizing GAG deposition. Positive staining is demarcated via blue arrows. (**B**) Polarized light images of picrosirius red stained sections demonstrating collagen production. Collagen is displayed orange under polarized light. All images taken with 10× objective on a Leica microscope. (Scale bar = 100 µm).

Lastly, when looking at the safranin-O (Figure 9A) and picrosirius red (Figure 9B) stains of the sectioned cell-laden constructs, it can be seen that in vitro culture in the chondrogenic medium had a positive effect on the cartilaginous matrix production of ATDC5 cells encapsulated in the 3D PEG hydrogels. More specifically, safranin-O stains for highly-sulfated GAGs [54], and thus the images should qualitatively correspond with the quantitative GAG assay. At 0 weeks, there was no indication of positive stain within any of the constructs. Although the image of F0 might seem to be depicting positive staining, it is due to a condensation artifact caused during the mounting process. At one week, both of the hydrogels with the R cross-linker displayed a higher amount of positive staining, depicted by the blue arrows, than the hydrogels formed with the F cross-linker. Similarly at four weeks, there were continued signs of positive safranin-O staining, especially in the hydrogel with the R cross-linker and RGD (6.5RR). The picrosirius red staining displayed similar trends as the safranin-O staining. Picrosirius red stains collagen, and due to its birefringence, the stained collagen will show up orange in color when imaged with polarized light [55,56]. For instance, at 0 weeks, none of the hydrogels depicted any positive collagen staining. At one week, some of the hydrogels, such as the RGD containing hydrogel with the regular degrading cross-linker (6.5RR), demonstrated a faint sign of positive orange spots. After four weeks, cells in all of the hydrogels displayed positive collagen staining, depicted by the orange spots spread out within the hydrogel.

Both sets of the stained histological sections depicted a more pericellular, localized staining, as opposed to being distributed throughout the hydrogel. This could be due to the properties of the hydrogels, such as mesh size, leading to the retention of the newly synthesized ECM components in a more local spot and preventing them from diffusing throughout the hydrogels. A similar phenomenon was observed with ATDC5 cells encapsulated in fibrin hydrogels (unpublished data). Additionally, when porcine vocal fold fibroblasts where encapsulated within unmodified PEG diacrylate hydrogels, a similar localized ECM deposition was observed, which was attributed to the unmodified nature of the hydrogel [46].

## 3. Materials and Methods

### 3.1. hPDC Cell Culture

Cells were harvested and isolated from similarly aged donors (*n* = 4; two male and two female donors, average age 13.7 ± 2.5 years) and combined together with respect to their in vitro growth kinetics and in vivo bone-forming capacity, as previously described [57]. Thawed hPDCs were seeded at an initial density of 5000 cells/cm^2^ in T-150 tissue flasks and cultured in growth medium (GM) composed of high-glucose Dulbecco’s Modified Eagle’s Medium (DMEM Glutamax; Invitrogen, Merelbeke, Belgium), supplemented with 10% fetal bovine serum (FBS; BioWhittaker, Walkersville, MD, USA), 1% antibiotic–antimycotic solution (100 units/mL penicillin; 100 µg/mL streptomycin; 0.25 µg/mL amphoterecin B; Invitrogen), and 1% sodium pyruvate (1 mM; Invitrogen). Cells at passage 8 were trypsin-released and encapsulated within the PEG hydrogels. Informed consent was obtained from each of the donors, and all procedures involving primary cells were approved by the Medical Ethics Committee UZ KU Leuven (study reference S53717, approved on 8 October 2012).

### 3.2. ATDC5 Cell Culture

ATDC5 cells (Riken Cell Bank, Tsukuba, Japan) were grown in a maintenance medium (MM) composed of Dulbecco’s Modified Eagle Medium/Ham’s Nutrient Mixture F12 (DMEM/F12) (1:1; Invitrogen), 5% fetal bovine serum (FBS; BioWhittaker), 1% antibiotic–antimycotic solution, 10 mg/mL human transferrin (Sigma, Bornem, Belgium), and 30 nM sodium selenite (Sigma), as previously described [33,36]. Medium was refreshed every 2 to 3 days, and the cells were passaged prior to becoming confluent.

### 3.3. Hydrogel Preparation and Cell Encapsulation

Michael-Type addition-based PEG-VS hydrogels-containing cells were made via the protocol previously described [7]. Briefly, for the initial in vitro proliferation and viability screening with hPDCs, 20 kDa 4-arm PEG-VS (JenKem, Plano, TX, USA) was dissolved in 0.3 M HEPES buffer, pH 8, for final concentrations of 2.5%, 4%, 6.5%, or 8% (*w*/*v*). For the DoE based experiments, only 4 and 6.5% (*w*/*v*) were utilized. For the in vitro chondrogenic screening studies with ATDC5 cells and hPDCs, a final concentration of only 6.5% (*w*/*v*) was used. Subsequently, 150 µM (final concentration in hydrogel) of the adhesion binding peptide containing a free thiol (Ac-GCGYGRGDSPG-NH_2_) was functionalized onto the PEG-VS macromer. For hydrogels without RGD (0 µM), an equal volume of buffer was added. Following an incubation period, a trypsin released cell suspension of either hPDCs for proliferation studies (1 × 10^6^ cells/ml final concentration) or hPDCs and ATDC5 cells for differentiation studies (10 × 10^6^ cells/ml final concentration) was mixed with two different dithiol, MMP-sensitive cross-linkers with different degradation rates, referred to as either R or F (Ac-GCREGPQGIWGQERCG-NH_2_ or Ac-GCRDVPMSMRGGDRCG-NH_2_, respectively [19]). Finally, the PEG-VS solutions, with or without RGD incorporation, were reacted with their respective cross-linker and cell suspensions. Additionally, as a control for the incorporation of the cell binding sequence, a scrambled RDG sequence was alternatively incorporated into some of the hydrogels. The hydrogel nomenclature indicates the concentration of PEG, type of cross-linker, and presence or absence of RGD (Table 1). Twenty microliter drops of the hydrogel precursor solutions were sandwiched between hydrophobic SigmaCote (Sigma) treated glass slides with 1 mm spacers and incubated at 37 °C for 30 min to allow full cross-linking to occur. The resulting cell-laden hydrogel disks of approximately 8 mm in diameter were cultured in well plates with the respective media formulations. Peptides were sourced from Biomatik (Cambridge, ON, Canada) or PeptideSynthetics (Fareham, UK) or were prepared by solid phase peptide synthesis and purified via HPLC-MS.

### 3.4. Culture Conditions

For the proliferation studies with hPDCs, cell-laden hydrogels were cultured in well plates in regular GM. Samples were analyzed at 1 week and 4 weeks of culture. For the differentiation studies, the cell-laden constructs were placed in well plates with GM and MM for hPDCs and ATDC5s, respectively. After 24 h, all the cell-laden constructs were switched to 4C chondrogenic differentiation medium (DMEM/F12 (Invitrogen) with 5% FBS, 10 ng/ml transforming growth factor-β1 (PeproTech), 1X Insulin Transferrin Selenium + premix (ITS+; BD Biosciences, Erembodegem, Belgium), 100 µg/mL ascorbic acid, 100 nM dexamethasone, and 1% antibiotic–antimycotic) [33,35], which was replenished every 2 to 3 days. Samples were analyzed prior to switching to chondrogenic differentiation medium (defined as 0 weeks) and after 1 and 4 weeks in the chondrogenic differentiation medium.

### 3.5. Cell Viability Assay

The capability of the different hydrogel compositions to support cellular viability was analyzed using a Live/Dead kit (Invitrogen). After a wash with phosphate buffered saline (PBS; Gibco, Merelbeke, Belgium) for 5 to 10 min, a staining solution of calcein AM and ethidium homodimer-1 (ETH-1) in PBS was added to the wells and incubated for 30 to 45 min at 37 °C. Following the incubation, the constructs were further washed in PBS. The stained samples were imaged using a FluoView confocal microscope with 10× objective at step size of 10 μm and a total thickness of 500 μm (Olympus, Berchem, Belgium). The 3D view in the Imaris software 7.2.3 (Bitplane, Zurich, Switzerland) was utilized to quantify the images. The images are represented as the maximum intensity of the z-stack in all figures.

### 3.6. PicoGreen/Quant-iT DNA Quantification

A PicoGreen/Quant-iT kit (Invitrogen) was used to investigate the effect of the various hydrogel formulations on cellular proliferation. The DNA content of three hydrogels per condition and per time point was calculated. The hydrogels were first digested in a buffer composed of 0.5 mg/mL Proteinase-K (Sigma) and 0.1% Triton X-100 in 5 mM EDTA in PBS, pH 7.1 (PBE) overnight at 60 °C. Following the complete degradation of the constructs, a working solution of the provided reagent was prepared according to the manufacturer’s instructions. The samples were read using the Qubit Fluorometer (Invitrogen), and the DNA concentration was calculated based on the equation provided by the manufacturer.

### 3.7. Design of Experiments Analysis

A ‘Design of Experiments’ (DoE) analysis was utilized to help determine the influence that the different variables of the hydrogel composition had on the proliferation of the cells. A three factor (PEG % (*w*/*v*), MMP sensitive peptide cross-linker, and RGD concentration (µM)), two-level fractional factorial design was used. The 4% and 6.5% (*w*/*v*) PEG concentrations were selected based on the initial proliferation screening criteria. The two cross-linkers with different degradation rates (R vs. F) were identified based on previous work [19]. The RGD concentrations were as previously described, 0 vs. 150 µM. A JMP software package 10 (SAS, Marlow, UK) was used to select a limited number of hydrogel formulations to evaluate further in the preliminary chondrogenic differentiation experiment.

### 3.8. DMMB GAG Assay

To quantify the extent of the chondrogenic differentiation taking place within the cell-laden hydrogels, a dimethylmethylene blue (DMMB) GAG assay was used. The samples digested by proteinase K that were used in the DNA assay were also used for the DMMB assay to quantify the amount of GAG produced, which was then normalized to the DNA content. Briefly, 1,9-dimethylmethylene blue chloride (Sigma) was dissolved in ethanol overnight and then added to reach a final concentration of 46 µM DMMB in a 0.04 M NaCl/glycine solution, pH 3. The filtered solution was measured to have 0.314 OD at 525 nm. Then, a serial dilution of chondroitin sulfate (CS; Sigma), ranging from 0 to 100 µg/mL, was prepared using PBE. Finally, 270 μL of the DMMB dye solution was combined with 30 μL of the samples or the CS standards in a 96-well plate, and the absorbance was read at 570 nm. The GAG concentration was calculated using the CS standard curve and subsequently divided by its corresponding DNA content to calculate GAG/DNA (µg/µg).

### 3.9. Histology

For the differentiation screening, hydrogels were first washed with two consecutive 10 min washes of fresh PBS. Following the PBS washes, the cell-laden constructs were placed in cassette sample holders and submerged in optimal cutting temperature (OCT) solution for 30 min. Subsequently, samples were snap frozen in liquid nitrogen, and 10-µm slices were sectioned on a cryostat machine and stained for safranin-O and picrosirius red to analyze the GAG production and collagen production, respectively. Briefly, for safranin-O staining, frozen sections were first let to air dry, rehydrated, and then immersed in 0.25% safranin-O solution for 7 min. Sections were then rinsed with water, fixed in methanol, dehydrated via graded ethanol, and mounted. For picrosirius red staining, air dried sections were rehydrated and placed in a picrosirius red solution (Sigma) for 1 h at room temperature. Next, stained sections were washed twice in fresh acidified water, fixed in methanol, dehydrated in graded ethanol, and finally, mounted. Brightfield images of safranin-O and polarized images of picrosirius red stained sections were taken using a DMR microscope (Leica Microsystems, Diegem, Belgium). For all sections, images were taken using a 10× objective.

### 3.10. Statistical Analysis

All quantitative outcomes (*n* = 3) are depicted as mean ± standard deviation. For the initial DNA quantification screening, the statistical analysis was done using a basic Student’s *t*-test to evaluate the effect of time among the hydrogels with similar compositions. An additional Student’s *t*-test was done between the hydrogels that were otherwise similar but differed in the RGD concentrations. A basic Student’s *t*-test was also conducted to compare the GAG production of the selected hydrogel formulations from the DoE. A two-way ANOVA was used for the subsequent DNA and GAG assays with hydrogel composition and time as the variables. Significant results and the respective *p*-values are indicated within each figure caption.

## 4. Conclusions

Combining the results of the ATDC5 cells with those of the hPDCs, the work herein has displayed that varying the composition of the hydrogels has different effects on the cells. From the initial proliferation study, it was evident that the 2.5% and 8% (*w*/*v*) constructs negatively affected cell survival, while the 4% and 6.5% (*w*/*v*) hydrogels helped cells proliferate over time, especially in the RGD containing compositions. We utilized the proliferation results as input criteria for the DoE to help further screen the different combinations. The DoE based chondrogenic differentiation screening helped to take one step closer to determining an ideal composition of the hydrogel constructs. In the DoE-related chondrogenic screening, the hydrogel made of 6.5% (*w*/*v*) macromer functionalized with RGD and the R cross-linker displayed the highest GAG/DNA after four weeks of in vitro culture. This was the same construct that also displayed one of the highest levels of DNA content in the proliferation experiment. Following the hPDC screening, the 6.5% (*w*/*v*) hydrogels were used to encapsulate ATDC5 cells, which were analyzed after culture in chondrogenic medium via the DMMB assay as well as qualitatively via safranin-O and picrosirius red histology. Taken together, via the screening studies, the cell-laden constructs prepared with 6.5% (*w*/*v*) PEG-VS macromer and cross-linked with the protease sensitive peptide cross-linkers can support both the proliferation and chondrogenic differentiation of these cell types. Therefore, these formulations would be interesting to further explore with different chondrogenic stimulation media to evaluate their effects on encapsulated cells. Furthermore, since ATDC5 cells have been shown to be the gold standard for in vitro chondrogenic simulation and testing, future work combining them with optimized hydrogels could be applied in research areas such as cartilage tissue on a chip [58].

## Figures and Tables

**Table 1:** Hydrogel nomenclature based on respective compositions.

Nomenclature	% (*w*/*v*) PEG	Cross-linker	RGD (µM)
2.5R0	2.5	Regular	0
2.5RR	2.5	Regular	150
2.5F0	2.5	Fast	0
2.5FR	2.5	Fast	150
4R0	4	Regular	0
4RR	4	Regular	150
4F0	4	Fast	0
4FR	4	Fast	150
6.5R0	6.5	Regular	0
6.5RR	6.5	Regular	150
6.5F0	6.5	Fast	0
6.5FR	6.5	Fast	150
8R0	8	Regular	0
8RR	8	Regular	150
8F0	8	Fast	0
8FR	8	Fast	150
6.5RD	6.5	Regular	150 (scrambled RDG)

## Abbreviations

ACI	Autologous chondrocyte implantation
CS	Chondroitin sulfate
DMEM	Dulbecco’s modified Eagle’s medium
DMMB	Dimethylmethylene blue
DoE	Design of experiments
DTT	Dithiothreitol
ECM	Extracellular matrix
ETH-1	Ethidium homodimer-1
FBS	Fetal bovine serum
GAG	Glycosaminoglycan
GM	Growth medium
hPDC	Human periosteum-derived cell
Ihh	Indian hedgehog
ITS+	Insulin transferrin selenium +
*k*_cat_	Catalytic rate constant
*K*_M_	Michaelis constant
MM	Maintenance medium
MMP	Matrix metalloproteinase
MSC	Mesenchymal stem cell
*M*_w_	Molecular weight
OCT	Optimal cutting temperature
PAMPS	Poly(2-acrylamido-2-methyl-1-propanesulfonic acid)
PBE	Phosphate buffered EDTA
PBS	Phosphate buffered saline
PDMAAm	Poly(dimethylacrylamide)
PEG	Polyethylene glycol
PEG-DM	Polyethylene glyocol di-methacrylate
PEG-VS	Vinyl sulfone end-functionalized polyethylene glycol
*pKa*	Dissociation constant
PTH	Parathyroid hormone
PTHrP	Parathyroid hormone receptor protein
